# Supplementary material for: Testing Denmark: a Danish Nationwide Surveillance Study of COVID-19
Source: Microbiol Spectr. 2021 Dec 15;9(3):e01330-21. doi: 10.1128/Spectrum.01330-21 (PMC8672904; doi:10.1128/Spectrum.01330-21)
Supplement: SUPPLEMENTAL FILE 1 — Supplemental material. Download SPECTRUM01330-21_Supp_1_seq7.pdf, PDF file, 1.1 MB [file spectrum01330-21_supp_1_seq7.pdf]

Supplementary materials:

Supplementary Table 1: In house validation (cases=600 individuals, controls=150 individuals)

Supplementary Table 2: Sex and age group distribution and place of living for responders and non-responders among population invited to participate in the questionnaire. Missing data represents invited persons who requested to be removed. Also shown are sex and age group distribution and place of living for participants who provided POCT results versus participants who did not provide the POCT results.

Supplementary Figure 1: Age and sex distribution of the final study population. Numbers above bars represent number of participants in each group.

Supplementary Figure 2: Distribution of SARS-CoV-2 antibodies according to age groups. Numbers above bars represent percentage of total number of participants in groups.

Supplementary Figure 3: SARS-CoV-2 seropositive % among 318,552 individuals stratified for age groups and sex. Numbers above bars represent total of participants within each group.

Supplementary Figure 4: SARS-CoV-2 seropositive % according to smoking habits, weekly alcohol consumption, and BMI stratified for sex. Red bar represents females, blue bar represents males. Numbers above bars represent number of participants in each group. P-values above bars represent chi-squared comparisons of males and females within groups. For panel B, the "Missing"-group encompasses participants who did not fill out weekly alcohol consumption in the questionnaire. For panel C, underweight, normal weight, pre-obesity and obese corresponds to BMI < 18.5, > 18.5 to 25, > 25 to 30, > 30.

Supplementary Figure 5: Seropositive % for self-assed risk of being infected and seropositive % for participants with a previous positive PCR test. Purple bar represents a subset of 1,828 participants with a positive PCR test prior to POCT test. Red, green, and blue bars represent participants without a prior positive PCR test. The self-assed risk of being infected was compared to the result of POCT test

Supplementary Figure 6: Flowchart for identifying participants with a positive PCR test before POCT.

Supplementary Figure 7: SARS-Cov-2 seropositive % among 804 individuals with an available date of POCT and available date of PCR test. The seroprevalence is strafied for days since positive.

Supplementary Figure 8: Map of seropositivity for each municipality.

Supplementary Figure 9: SARS-CoV-2 seropositive % in households with a COVID-19 infected person stratified by household size. Numbers above bars represent number of seropositive and seronegative participants in each household size.

Supplementary Figure 10: Proportion of persons who experienced symptoms stratified for age groups among all participants.. Numbers next to bars represent percentages. Numbers in facet labels represent total participants within each age group.

Supplementary Figure 11: Number of previous tests for 318,552 participants stratified for age groups and sex.

Supplemental text: The questionnaire

Supplementary Table 1: In house validation (cases=600 individuals, controls=150 individuals)

**LIVZON**					
**Batchnummer**	**Expiration date for POCT**	**Sensitivity**	**95% CI**	**Specificity**	**95% CI**
CK2004310410	14.10.2020	93.3%	88.1-96.7	98.2%	96.7-99.1
CK2004350410	19.10.2020	92.7%	87.3-96.3	97.5%	95.9-98.6

Supplementary Table 2: Sex and age group distribution and place of living for responders and non-responders among population invited to participate in the questionnaire. Missing data represents invited persons who requested to be removed. Also shown are sex and age group distribution and place of living for participants who provided POCT results versus participants who did not provide the POCT results.

Col: Column, POCT: Point of care test

	**Answered Questionnaire**			**Provided POCT result**		
**N<br>Row %<br>Col %**	**No**	**Yes**	**Total**	**No**	**Yes**	**Total**
**Females**	384,507<br>58.6 %<br>46.6 %	272,012<br>41.4 %<br>57.3 %	656,519<br>50.5 %	43,538<br>18.9 %<br>54.4 %	186,342<br>81.1 %<br>57.9 %	229,880<br>57.2 %
**Males**	440,939<br>68.5 %<br>53.4 %	202,399<br>31.6 %<br>42.8 %	643,338<br>49.5 %	36,433<br>21.2 %<br>45.6 %	135,789<br>78.9 %<br>42.2 %	172,222<br>42.8 %
**Total**	825,446<br>63.5 %	474,411<br>36.5 %	1,299,857<br>100.00	79,971<br>19.9 %	322,131<br>80.1 %	402,102<br>100 %
**Age group, N<br>Row %<br>Col %**	**No**	**Yes**	**Total**	**No**	**Yes**	**Total**
**15-25**	147,686<br>77.4 %<br>17.9 %	43,130<br>22.6 %<br>9.1 %	190,816<br>14.7%	10,122<br>28.7 %<br>12.7 %	25,112<br>71.3 %<br>7.8 %	35,234<br>8.8 %
**25-34**	141,758<br>69.3 %<br>17.2 %	62,679<br>30.7 %<br>13.2 %	204,437<br>15.7 %	15,014<br>27.9 %<br>18.8 %	38,831<br>72.1 %<br>12.1 %	53,845<br>13.4 %
**35-44**	113,045<br>62.2 %<br>13.7 %	68,857<br>37.9 %<br>14.5 %	181,902<br>14.0 %	14,382<br>24.1 %<br>18.0 %	45,212<br>75.9 %<br>14.0 %	59,594<br>14.8 %
**45-54**	116,215<br>54.6 %<br>14.1 %	96,645<br>45.4 %<br>20.4 %	212,860<br>16.4 %	16,488<br>19.8 %<br>20.6 %	66,803<br>80.2 %<br>20.7 %	83,291<br>20.7 %
**55-64**	99,021<br>50.1 %<br>12.0 %	98,632<br>49.9 %<br>20.8 %	197,653<br>15.2 %	13,581<br>16.1 %<br>17.0 %	70,831<br>83.9 %<br>22.0 %	84,412<br>21.0 %
**65-74**	94,985<br>55.7 %<br>11.5 %	75,551<br>44.3 %<br>15.9 %	170,536<br>13.1 %	7,332<br>11.6 %<br>9.2 %	56,041<br>88.4 %<br>17.4 %	63,373<br>15.8 %
**75+**	112,736<br>79.6 %<br>13.7 %	28,917<br>20.4 %<br>6.1 %	141,653<br>10.9 %	3,052<br>13.7 %<br>3.8 %	19,301<br>86.4 %<br>6.0 %	22,353<br>5.6 %
**Total**	825,446<br>63.5 %	474,411<br>37,0 %	1,299,857<br>100 %	79,971<br>19.89	322,131<br>80.11	402,102<br>100.0 %
**Place of living, N<br>Row %<br>Col %**	**No**	**Yes**	**Total**	**No**	**Yes**	**Total**
**The Capital Region of Denmark**	262,620<br>63.9 %<br>31.8 %	148,622<br>36.1 %<br>31.3 %	411,242<br>31.6 %	27,798<br>21.9 %<br>34.8 %	99,008<br>78.1 %<br>30.7 %	126,806<br>31.5 %
**Region Zealand**	121,320<br>64.6 %<br>14.7 %	66,595<br>35.4 %<br>14.0 %	187,915<br>14.5 %	11,779<br>21.0 %<br>14.7 %	44,334<br>79.0 %<br>13.8 %	56,113<br>14.0 %
**The Region of Southern Denmark**	174,432<br>63.9 %<br>21.1 %	98,359<br>36.1 %<br>20.7 %	272,791<br>21.0 %	15,338<br>18.5 %<br>19.2 %	67,761<br>81.5 %<br>21.0 %	83,099<br>20.7 %
**Central Denmark Region**	183,623<br>62.1 %<br>22.3 %	111,950<br>37.9 %<br>23.6 %	295,573<br>22.7 %	17,313<br>18.3 %<br>21.7 %	77,292<br>81.7 %<br>24.0 %	94,605<br>23.5 %

**The North Denmark Region**	83,451<br>63.1 %<br>10.1 %	48,885<br>36.9 %<br>10.3 %	132,336<br>10.2 %	7,743<br>18.7 %<br>9.7 %	33,736<br>81.3 %<br>10.5 %	41,479<br>10.3 %
**Total**	825,446<br>63.5 %	474,411<br>36.5 %	1,299,857<br>100 %	79,971<br>19.89	322,131<br>80.1 %	402,102<br>100.0 %
**Missing 21**						

Supplementary Figure 1: Age and sex distribution of the final study population. Numbers above bars represent number of participants in each group.

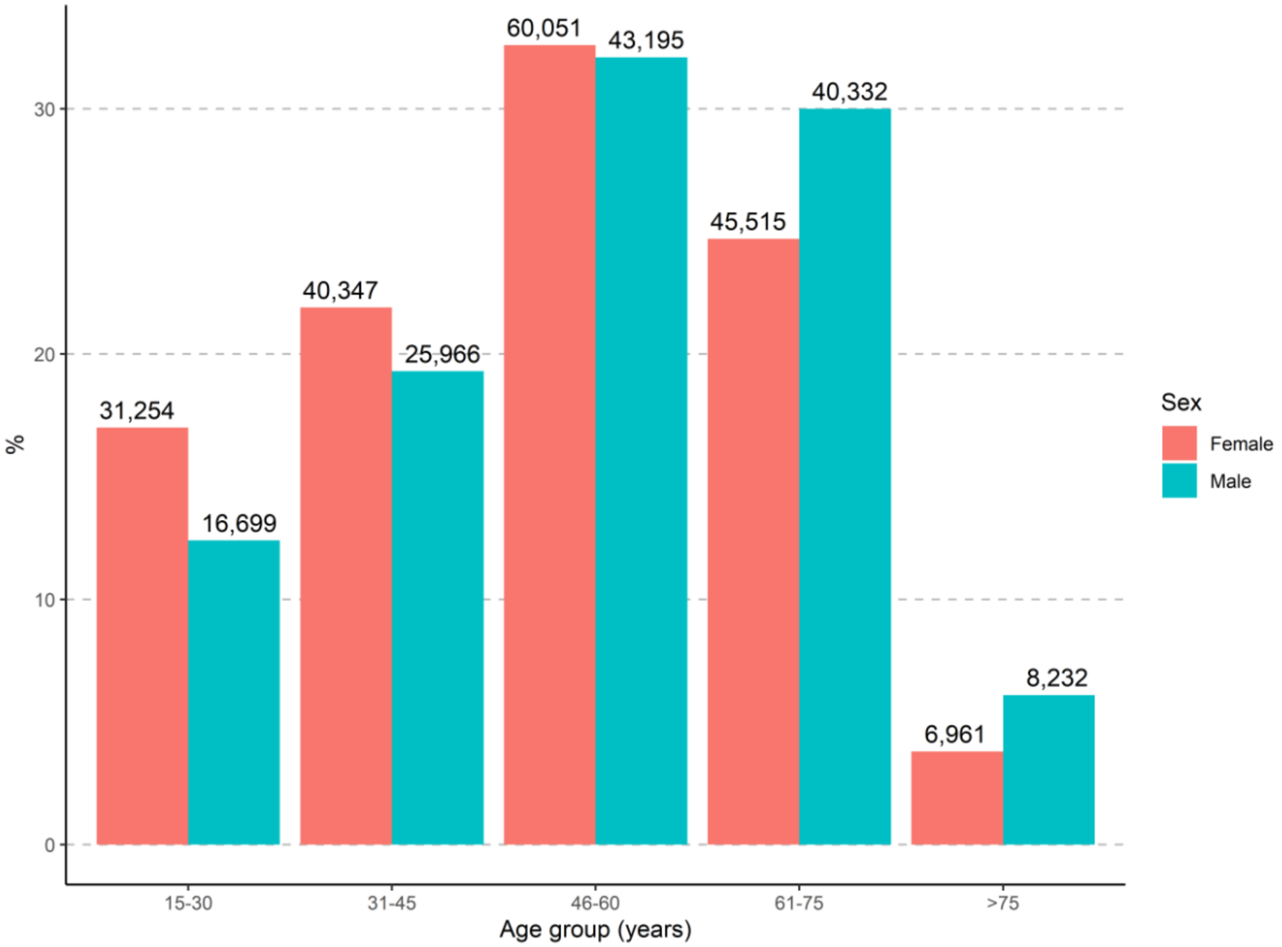

Supplementary Figure 2: Distribution of SARS-CoV-2 antibodies according to age groups. Numbers above bars represent percentage of total number of participants in groups.

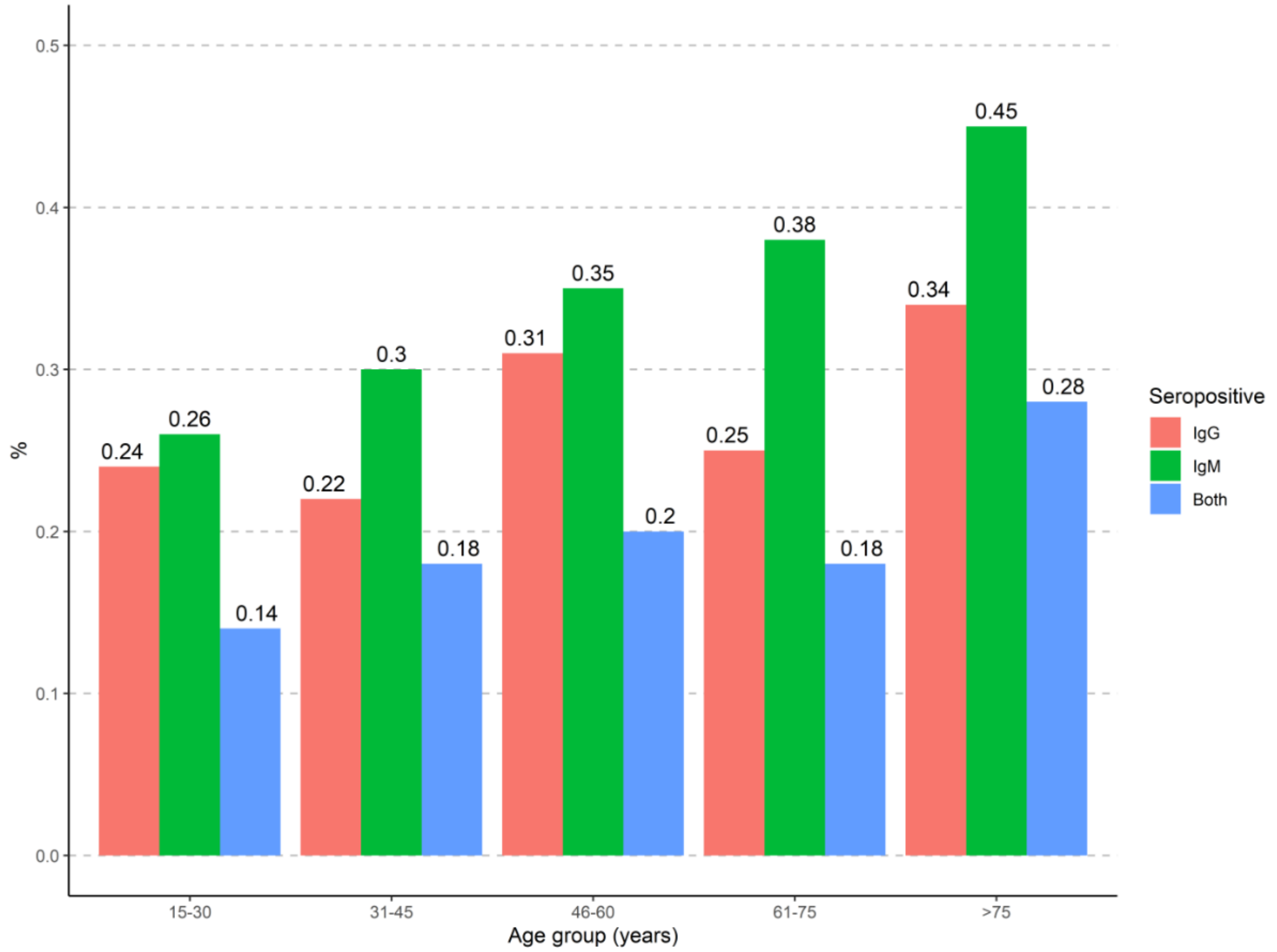

Supplementary Figure 3: SARS-CoV-2 seropositive % among 318,552 individuals stratified for age groups and sex. Numbers above bars represent total of participants within each group.

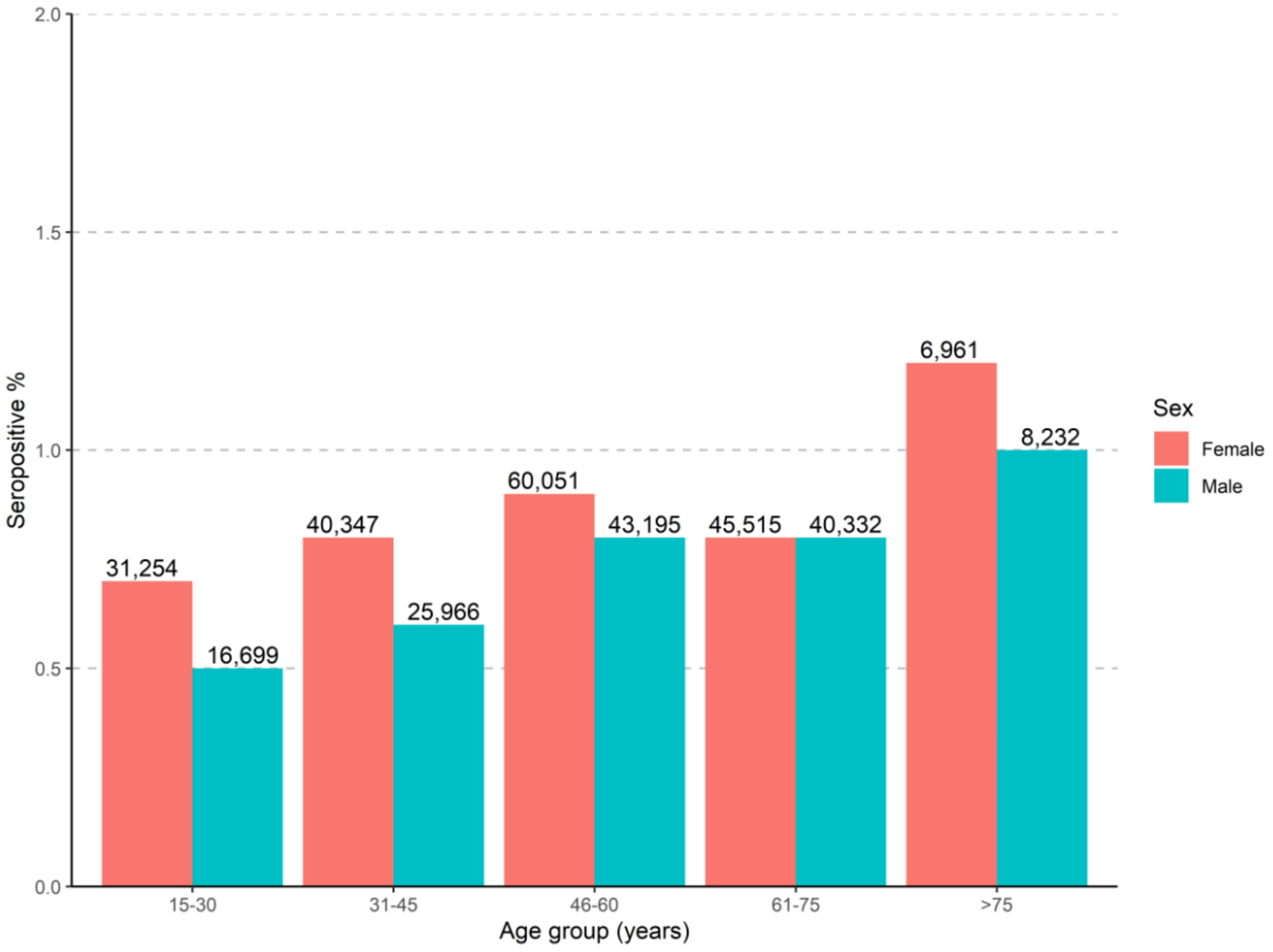

Red: female (n, 148,128), blue: male (n, 134,424), number of participants in each group

Supplementary Figure 4: SARS-CoV-2 seropositive % according to smoking habits, weekly alcohol consumption, and BMI stratified for sex. Red bar represents females, blue bar represents males. Numbers above bars represent number of participants in each group. P-values above bars represent chi-squared comparisons of males and females within groups. For panel B, the "Missing"-group encompasses participants who did not fill out weekly alcohol consumption in the questionnaire. For panel C, underweight, normal weight, pre-obesity and obese corresponds to BMI < 18.5, > 18.5 to 25, > 25 to 30, > 30.

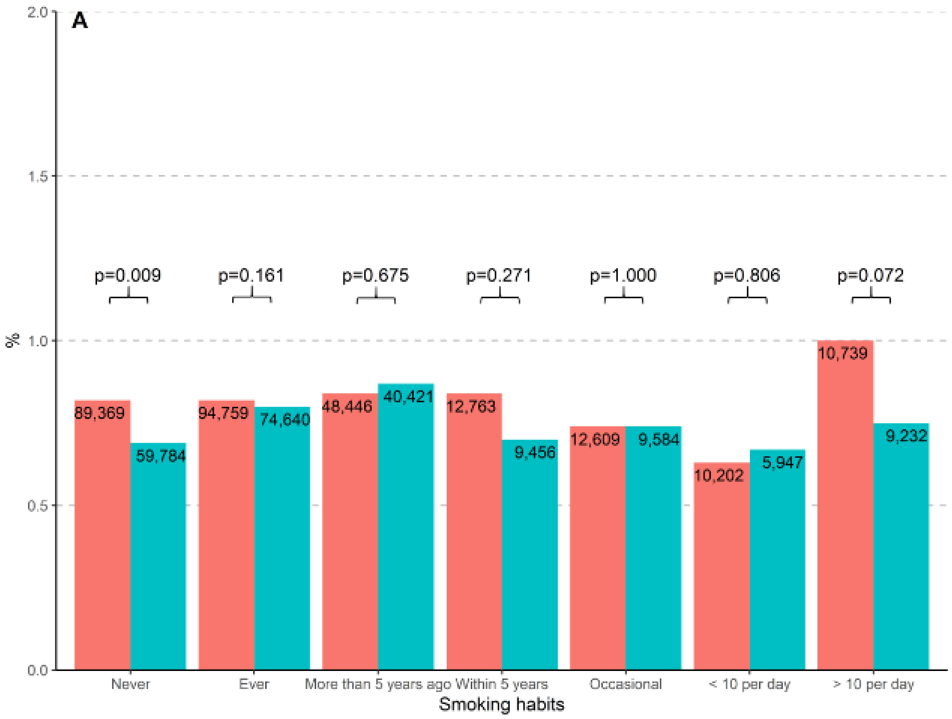

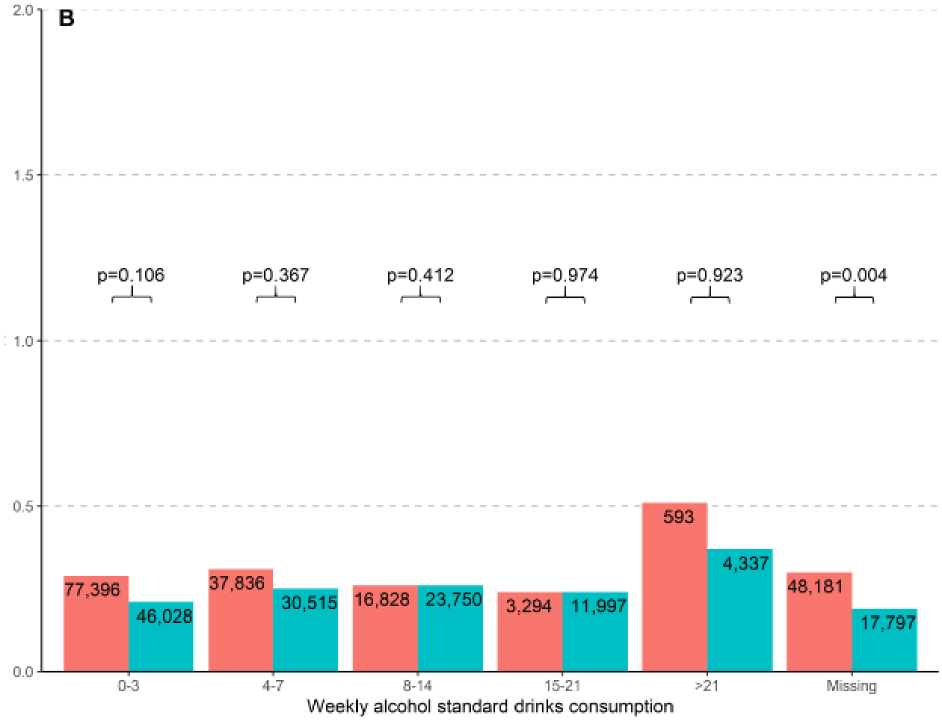
B
2.0
1.5
1.0
0.5
0.0
p=0.106
p=0.367
p=0.412
p=0.974
p=0.923
p=0.004
77,396
46,028
37,836
30,515
16,828
23,750
3,294
11,997
593
4,337
48,181
17,797
0-3
4-7
8-14
15-21
>21
Missing
Weekly alcohol standard drinks consumption

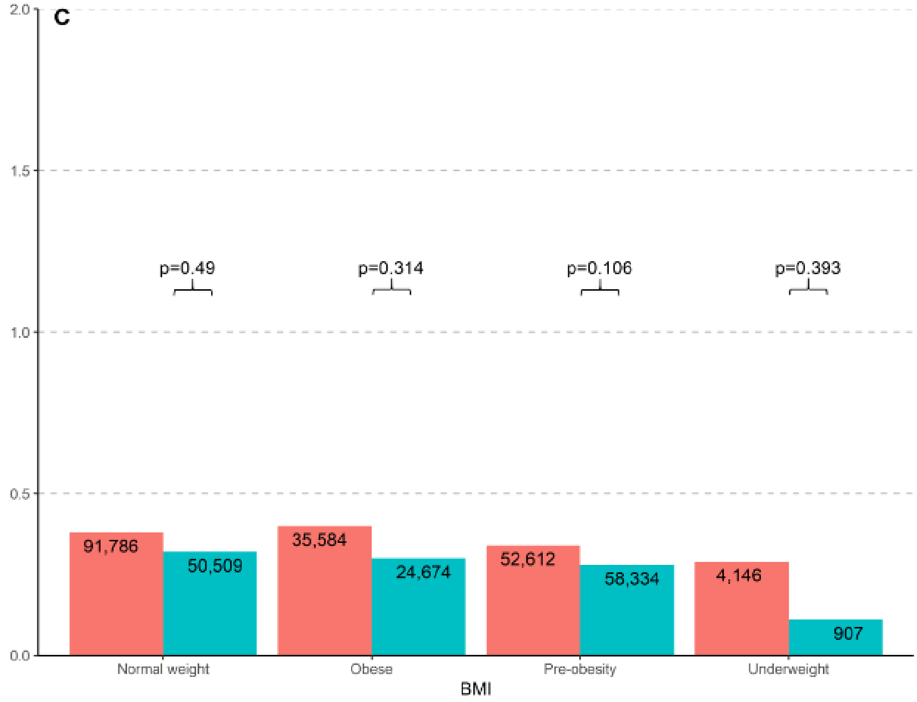
C
2.0
1.5
1.0
0.5
0.0
p=0.49
p=0.314
p=0.106
p=0.393
91,786
50,509
35,584
24,674
52,612
58,334
4,146
907
Normal weight
Obese
Pre-obesity
Underweight
BMI

Supplementary Figure 5: Seropositive % for self-assed risk of being infected and seropositive % for participants with a previous positive PCR test. Purple bar represents a subset of 1,828 participants with a positive PCR test prior to POCT test. Red, green, and blue bars represent participants without a prior positive PCR test. The self-assed risk of being infected was compared to the result of POCT test.

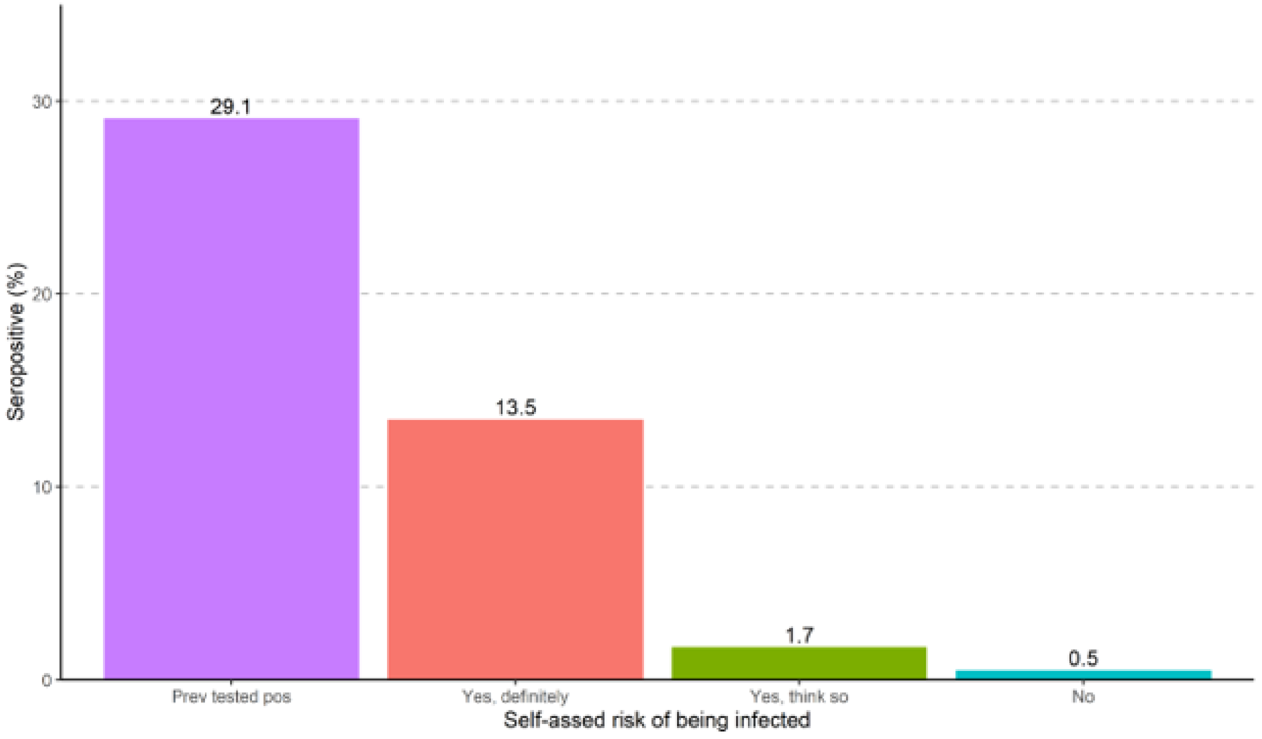

Supplementary Figure 6: Flowchart for identifying participants with a positive PCR test before POCT.

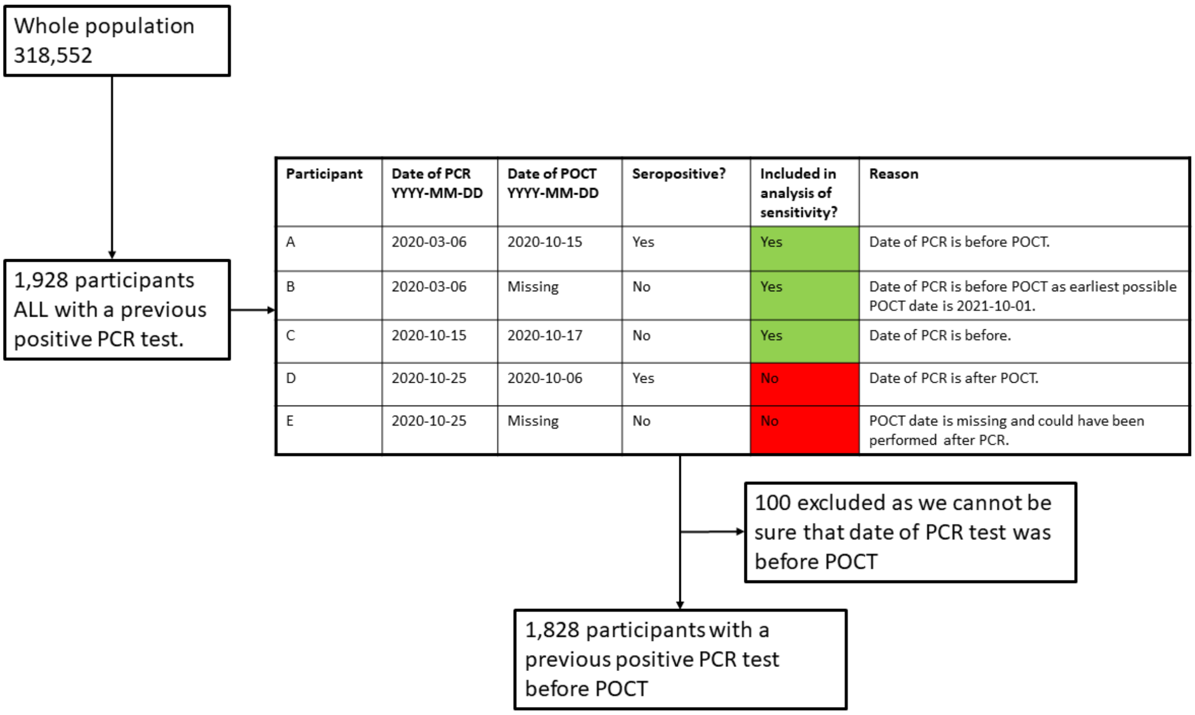

Participant	Date of PCR YYYY-MM-DD	Date of POCT YYYY-MM-DD	Seropositive?	Included in analysis of sensitivity?	Reason
A	2020-03-06	2020-10-15	Yes	Yes	Date of PCR is before POCT.
B	2020-03-06	Missing	No	Yes	Date of PCR is before POCT as earliest possible POCT date is 2021-10-01.
C	2020-10-15	2020-10-17	No	Yes	Date of PCR is before.
D	2020-10-25	2020-10-06	Yes	No	Date of PCR is after POCT.
E	2020-10-25	Missing	No	No	POCT date is missing and could have been performed after PCR.

Supplementary Figure 7: SARS-Cov-2 seropositive % among 804 individuals with an available date of POCT and available date of PCR test. The seroprevalence is strafied for days since positive.

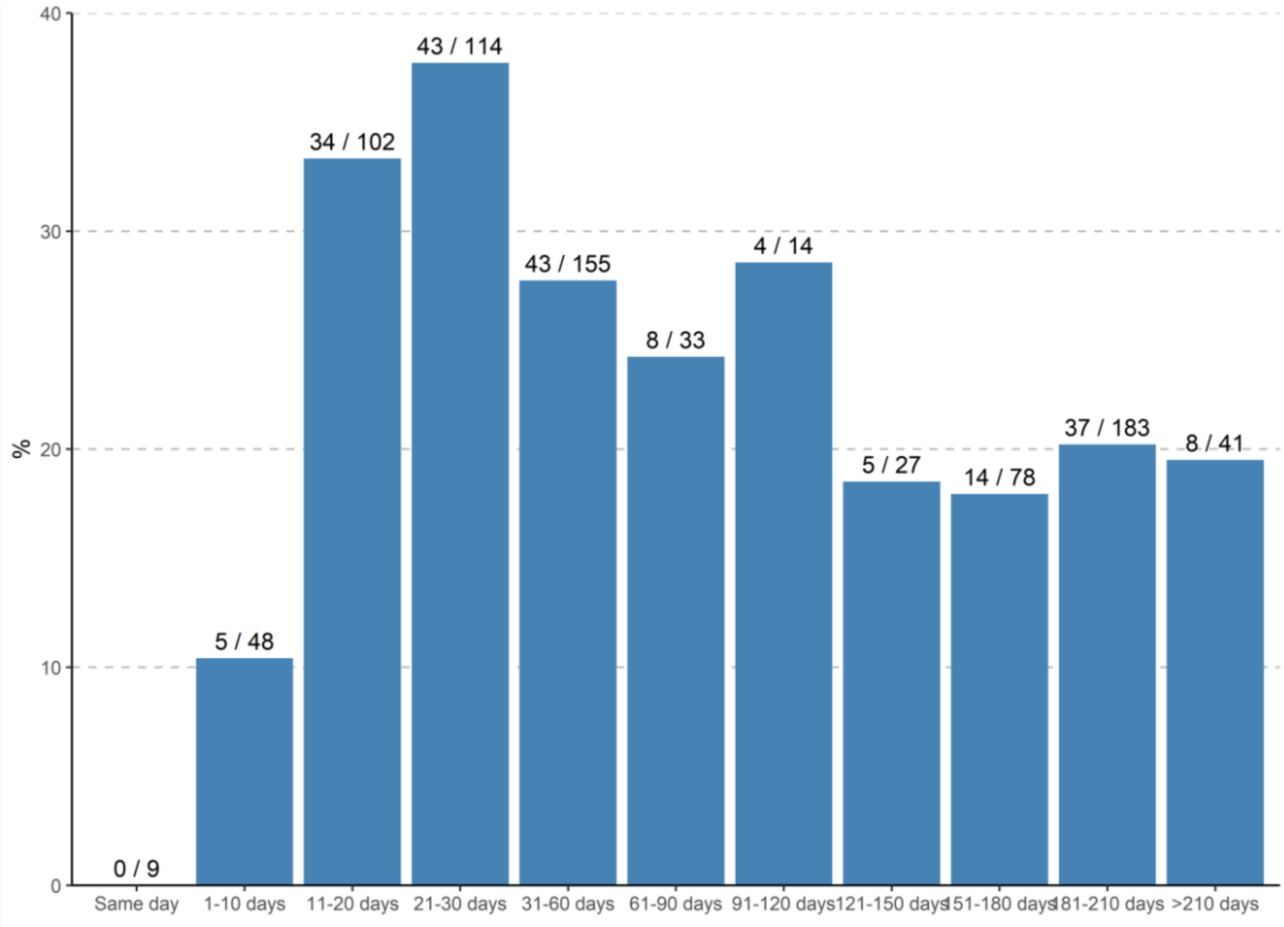

Supplementary Figure 8: Map of seropositivity for each municipality.

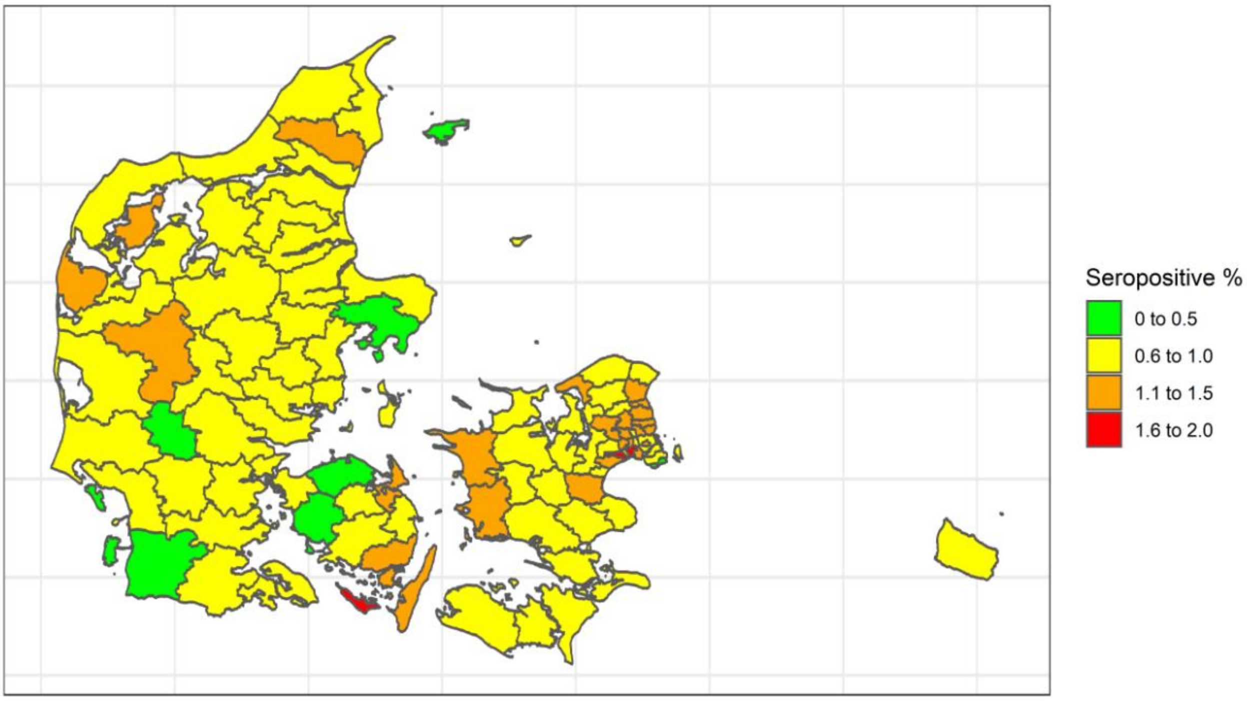

Supplementary Figure 9: SARS-CoV-2 seropositive % in households with a COVID-19 infected person stratified by household size. Numbers above bars represent number of seropositive and seronegative participants in each household size.

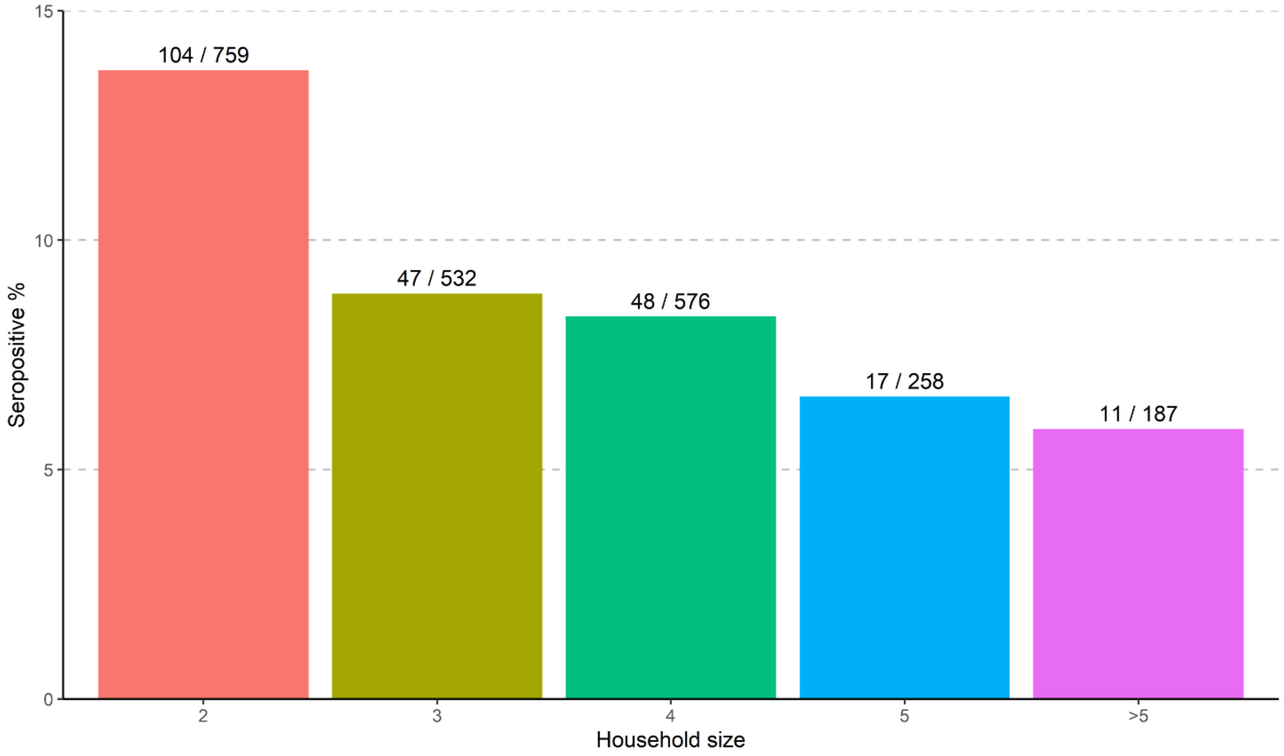

Supplementary Figure 10: Proportion of persons who experienced symptoms stratified for age groups among all participants. Numbers next to bars represent percentages. Numbers in facet labels represent the total number of participants within each age group.

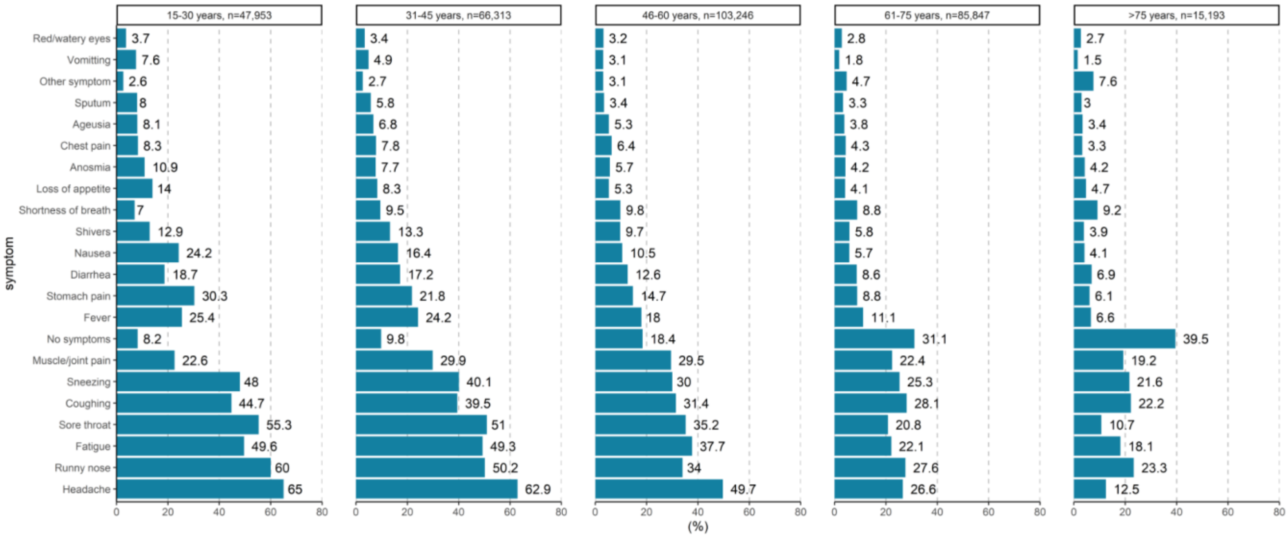

Supplementary Figure 11: Number of previous tests for 318,552 participants stratified for age groups and sex.

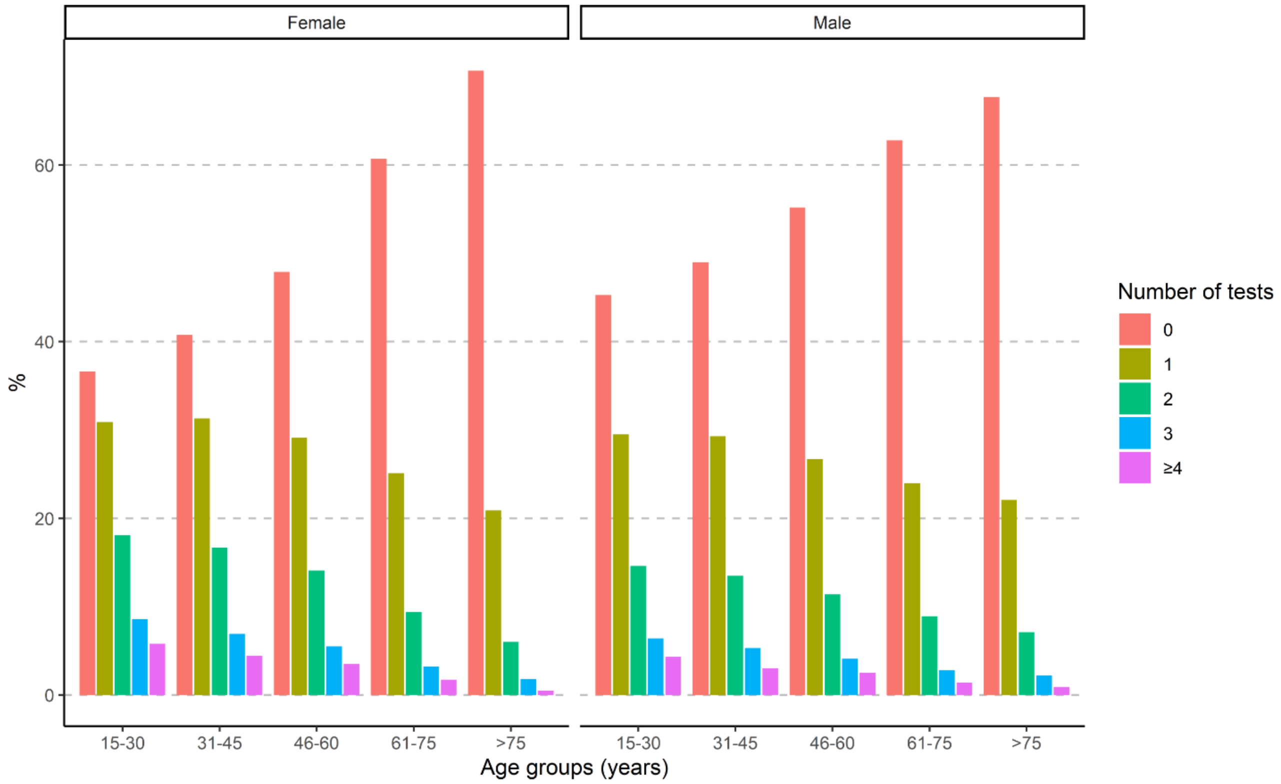

Supplemental text: The questionnaire

1. Testing Denmark NOTE it is no longer possible to sign up for the antibody testing. It will still to be very valuable to us if you will take the time to fill out the questionnaire.
2. Questions about infection with COVID-19
3. If you were to give your best bet, do you think/know that you have ever been infected with COVID-19?
4. Have you previously been tested for COVID-19?
5. How many times have you been tested for COVID-19?
6. What type of COVID-19 test have you had? (Feel free to give multiple answers)
7. Was one or more of your tests positive?
8. Symptoms
9. How often do you usually have a cold or influenza-like symptoms?
10. Have you had any of the following symptoms in the period since 1 February 2020?
11. How many times have you had a fever since 1 February 2020?
12. How many times have you had chills since 1 February 2020?
13. How many times have you had runny or stuffy nose since 1 February 2020?
14. How many times have you had an impaired sense of smell since 1 February 2020?
15. How many times have you had an impaired sense of taste since 1 February 2020?
16. How many times have you had periods with sneeze since 1 February 2020?
17. How many times have you had a sore throat since 1 February 2020?
18. How many times have you had a cough since 1 February 2020?
19. How many times did you experience difficulty in breathing/shortness of breath since 1 February 2020?
20. How many times have you had a headache since 1 February 2020?
21. How many times have you experienced muscle and/or joint pain since 1 February 2020?
22. How many times have you had chest pain since 1 February 2020?
23. How many times have you experienced fatigue and exhaustion since 1 February 2020?
24. How many times have you experienced a loss of appetite since 1 February 2020?
25. How many times have you experienced coloured sputum (spit)/mucus since 1 February 2020?
26. How many times have you had bloodshot, watery eyes since February 1 2020?
27. How many times have you experienced nausea since 1 February 2020?
28. How many times have you vomited since 1 February 2020?
29. How many times have you had diarrhoea since 1 February 2020?
30. How many times have you had stomach pain since 1 February 2020?
31. How many times have you had other symptoms since 1 February 2020?
32. In which months since 1 February did you experience fever?
33. In which months since 1 February did you experience chills?
34. In which months since 1 February did you experience a runny or stuffy nose?
35. In which months since 1 February did you experience an impaired sense of smell?
36. In which months since 1 February did you experience an impaired sense of taste?
37. In which months since 1 February did you experience sneezing?
38. In which months since 1 February did you experience a sore throat?
39. In which months since 1 February did you experience a cough?
40. In which months since 1 February did you experience difficulty breathing?
41. In which months since 1 February did you experience headaches?
42. In which months since 1 February did you experience muscle and/or joint pains?
43. In which months since 1 February did you experience chest pains?
44. In which months since 1 February did you experience fatigue and exhaustion?
45. In which months since 1 February did you experience a loss of appetite?
46. In which months since 1 February did you experience coloured sputum (spit)/mucus?
47. In which months since 1 February did you experience bloodshot, watery eyes?
48. In which months since 1 February did you experience nausea?
49. In which months since 1 February did you experience vomiting?
50. In which months since 1 February did you experience diarrhoea?
51. In which months since 1 February did you experience stomach pains?
52. In which months since 1 February did you experience other symptoms?

53. Did you take your temperature when you had a fever?
54. What was your highest measured temperature?
55. Did your symptoms occur suddenly (over a few hours)?
56. Did you take any medication for your symptoms?
57. What do you think is/was the cause of your symptoms? (Feel free to give multiple answers)
58. Which of the following conditions best describes how you felt when you were feeling the worst, while you had/suspected you had COVID-19?
59. Risk of COVID-19
60. Since 1 February, have you at any time been in contact with/in proximity to someone whom you knew had tested positive for COVID-19? That is within seven days before or after this person tested positive for COVID-19?
61. Contact with infected
    61.1 Have you stayed for minimum 15 minutes in the same room as an infected person?
    61.2 Have you had body contact with a person infected with COVID-19?
    61.3 Have you worked/studied with someone who was infected with COVID-19?
    61.4 Has someone in your household been infected with COVID-19?
    61.5 Has someone in your family or a friend outside your household been infected with COVID-19?
62. Have you traveled abroad since 1 February 2020?
63. Approximately how many times have you traveled abroad (with sleepover) since 1 February 2020
64. Which of the following countries have you traveled to (with sleepover) since 1st of february (feel free to give multiple answers)
65. This part concerns behaviour
66. Have you taken any of the following measures in the past 14 days due to the risk of COVID-19 infection? (Feel free to give multiple answers)
67. Which of the statements below apply to you?
68. Have you consumed alcohol in the past 12 months?
69. On how many days a week do you drink alcohol on average?
70. How many units do you typically drink a week?
71. 1 unit =
72. Chronic illness. Information about any chronic illness, height, weight and lifestyle is important to enable us to assess whether you are at a particular risk of COVID-19.
73. For each of the following diseases and health problems, please state whether you currently suffer from it or have previously suffered from it.
    73.1 Asthma
    73.2 Allergy (other than asthma)
    73.3 Diabetes
    73.4 High blood pressure
    73.5 Heart attack
    73.6 Stroke
    73.7 Chronic bronchitis, hyperinflated (enlarged) lungs, smoker's lungs (emphysema, COPD)
    73.8 Rheumatoid arthritis
    73.9 Cancer
    73.10 Other chronic disease
74. What other chronic disease
75. How much do you weigh in kilograms (kg)?
76. How tall are you in centimetres (cm)?
77. Did you get an influenza vaccine last autumn/winter 2019-2020?
78. Work and education
79. What is your highest level of completed education?
80. What is your main occupation?
81. Which area(s) or type(s) of work best describe(s) your work? (Feel free to give multiple answers)
82. Are you in contact with patients in your work?
83. Have you worked with patients hospitalised with COVID-19?
84. The following questions concern your household
85. How many people in the following age groups, including yourself, live in your household?

85.1 0-4-year-olds
85.2 5-18-year-olds
85.3 19-44-year-olds
85.4 45-64-year-olds
85.5 65+-year-olds

86. The following questions concern your perception of your health
87. In general would you say your health is
88. How do you experience your health now relative to last year at the same time?
89. The following questions are about activities you might do during a typical day.
90. Does your health now limit you in these activities? If so, how much?
    90.1 Moderate activities, such as moving a table, pushing a vacuum cleaner or bicycling
    90.2 Climbing several flights of stairs
    90.3 Hard activities, such as sports, running or other hard physical activity
91. Have you had any of the following problems with your work or other day-to-day activities due to your physical health in the past four weeks?
    91.1 I've managed less than I would've liked to
    91.2 I've been restricted in the kind of work or other activities I've been able to perform
92. During the past 4 weeks, have you been limited in daily activities due to physical pain (including activities at home and at work)?
93. How do you experience your discomfort from pain now relative to last year at the same time?
94. How do you experience your physical health now relative to last year at the same time?
95. Have you had any of the following problems with your work or other day-to-day activities due to emotional problems in the past four weeks?
    95.1 I've managed less that I would've liked to
    95.2 I have performed my work or other activities less carefully than I usually do
96. How do you experience your emotional health now relative to last year at the same time?
97. These questions are about how you have felt in the past four weeks. How much of the time in the past four weeks have you
    97.1 ... felt calm and peaceful
    97.2 ... been full of energy
    97.3 ... felt downhearted and blue
98. Within the past four weeks, how much of the time has your physical health or emotional problems made it difficult for you to see other people (e.g. visit friends, relatives etc.)?
99. If you have elaborating comments on the questions, you can write them here
100. Special health problems now and a year ago
101. If you have obvious explanations for your problems yourself, you can state them in a box at the end. For each condition, please state how this has changed relative to last year -5 = Much worse 0 = The same 5 = Much better
102. Fatigue – Relative to last year
103. Dry cough – Relative to last year
104. Chest discomfort – Relative to last year
105. Shortness of breath/difficulty breathing when walking at a brisk pace or running – Relative to last year
106. Shortness of breath/difficulty in breathing when walking at an easy pace or light work, e.g. vacuuming or gardening – Relative to last year
107. Shortness of breath/difficulty breathing when talking – Relative to last year
108. Shortness of breath/difficulty breathing when resting – Relative to last year
109. Headache – Relative to last year
110. Dizziness – Relative to last year
111. Pain in muscles and joints – Relative to last year
112. Nausea – Relative to last year
113. Vomiting – Relative to last year
114. Constipation – Relative to last year
115. Diarrhoea – Relative to last year
116. Tingling sensations in hands or feet – Relative to last year
117. Difficulty concentrating – Relative to last year

118. Difficulty remembering things that have just happened (short-term memory) – Relative to last year
119. Difficulty remembering things that happened a long time ago (long-term memory) – Relative to last year
120. Sensitivity to light – Relative to last year
121. Sensitivity to sound – Relative to last year
122. You quickly become tired when looking at a screen (computer, iPad, TV and the like) – Relative to last year
123. Reduced or altered sense of taste – Relative to last year
124. Reduced or altered sense of smell – Relative to last year
125. Have you had an unintended change in your weight over the past year?
126. If you have elaborating comments on the questions, you can write them here
127. Your mood
128. During the past two weeks, have you had little interest or joy in doing things?
129. During the past two weeks, have you felt down, depressive or had a feeling of hopelessness?
130. How do you experience your mood now relative to last year at the same time?
131. If you have elaborating comments on the questions, you can write them here
132. Antibody test
133. Would you like to be sent a home test which you can use to test whether you have COVID-19 antibodies?
134. Thank you so much for participating in the survey!
135. Thank you so much for participating in the survey!
